# Perinatal Stress, Fatigue, Depressive Symptoms, and Immune Modulation in Late Pregnancy and One Month Postpartum

**DOI:** 10.1155/2014/652630

**Published:** 2014-01-22

**Authors:** C. Y. Cheng, R. H. Pickler

**Affiliations:** ^1^Chang Gung University of Science and Technology, Chiayi 61363, Taiwan; ^2^Cincinnati Children's Hospital Medical Center, Cincinnati, OH 45229, USA

## Abstract

Stress and fatigue are common complaints of pregnant and postpartum women as is depression. These symptoms may be related to immunomodulation. However, few studies have examined these relationships. The aim of this study was to examine the relationships among stress, fatigue, depression, and cytokines as markers of immune modulation in prenatal and postpartum women. Women completed questionnaires and gave blood samples during late pregnancy and again at 4–6 weeks postpartum. Blood was analyzed for cytokines as measures of immune modulation. Stress, fatigue, and depression were experienced at moderately high levels, with higher levels of fatigue and depression in the postpartum but higher stress in the prenatal period. Levels of several cytokines were increased in the postpartum over the prenatal period. Stress and depression were related in the prenatal period and stress, depression, and fatigue were related in the postpartum. While various cytokines were related to each other in both periods, only stress was related to MIP-1**β**, a cytokine that may be important for childbirth processes. More studies, especially longitudinal and interventional studies, are needed to increase our knowledge about etiology, patterns, symptoms, factors, and management of maternal distress. The search for reliable biomarkers for at-risk mothers remains a priority.

## 1. Introduction

Pregnancy and the transition to motherhood may cause stress for women that negatively affects their psychological health [1]. Maternal stress is associated with early labor onset, poor fetal development, and poor child motor and mental development [2–4]. Fatigue has been found to be related to stress [5], with pregnant women often reporting tiredness or fatigue (87.2% to 96.5) [6–8]. However, few studies have focused on prenatal fatigue. Among those few studies, mothers perceived a significantly higher level of fatigue in the evening than in the morning as well as more severe fatigue from the seventh to ninth month of pregnancy [9], although the increase in fatigue may start as early as 11 to 12 weeks of gestation [10]. Prenatal fatigue is also related to prenatal depression, anxiety [11, 12], and preterm birth [13] and is predictive of cesarean delivery [6]. In other words, maternal stress and fatigue have a similar impact on a woman's health and well-being during pregnancy.

Depression during pregnancy and the postpartum period is more widely studied than stress or fatigue. The prevalence of prenatal depression reported differs by the tools used. For example, depression was reported to be 7.4%, 12.8%, and 12.0% in the first, second, and third trimester, respectively, when using Edinburgh Postnatal Depression Scale, Beck Depression Inventory, or structured interviews [14]. However, when using the Center for Epidemiologic Study-Depression (CES-D) scale, the mean rate was 20.4% among women of various ethnicities during the entire pregnancy [15]. Despite differences in rates of reported incidence, it is clear that women with prenatal depression have higher rates of preterm delivery and preeclampsia as well as poor fetal and infant outcomes [16]. Additionally, the factors related to prenatal depressive symptoms are generally agreed upon and include a history of depression, perceived poor health, alcohol and cigarette use, low educational level, unemployment, and unmarried status [15, 17]. Moreover, black women have higher rates of prenatal depressive mood than white women [17]. Finally, studies have demonstrated that depressive symptoms are associated with levels of stress and number of stressful life events [18, 19] and pregnant women experience more stressful events than nonpregnant women [20].

In an attempt to understand the psychological components of maternal well-being, including stress, fatigue, and depression, during pregnancy and with an aim toward identifying biomarkers that may help to identify women who are at risk for poor pregnancy outcomes, researchers have attempted to link immune modulation to maternal well-being. It is well known that to maintain a successful pregnancy, a healthy immune system is needed. It is hypothesized that cytokines, major biomarkers of immune function, produced by T helper (Th) 2 cells predominate over the cytokines produced by Th1 cells during mid- and late pregnancy [21]. Although the differences in function of cytokines produced by Th1 and Th2 cells are not entirely clear, generally examples of Th1 cytokines include interleukin-2 (IL-2) and interferon gamma (IFN-*γ*) while IL-4, IL-5, IL-6, and IL-10 are considered Th2 cytokines. So, for example, compared to nonpregnant women, IFN-*γ* is lower in the third trimester [22], while IL-6 and IL-10 increase from early pregnancy to late pregnancy [23]. Other cytokines, such as IL-2, IL-12, and tumor necrosis factor alpha (TNF-*α*), remain stable throughout pregnancy [24]. Unfortunately, there is little consensus about the meaning associated with various cytokine levels during pregnancy. For example, compared to nonpregnant women, IL-6, IL-10, and IFN-*γ* in normal pregnancy were found to be higher in one study [25], lower in another study [26], but showed no differences in other studies [27–29]. Moreover, while some studies have reported an increase in IL-6 and IFN-*γ* from early pregnancy to late pregnancy [23, 24, 29] others reported a decrease or no significant changes in their values [26, 30, 31].

Additionally, while there is interest in understanding the relationship of immune modulat on to psychological or physical well-being during and after pregnancy, very few studies have been conducted on these relationships. So, while stress has been found to be associated with lower immunoglobulin G (Ig) production [32] and thus with reduced immune function, the relationships between stress and cytokine levels are not consistent. Of the few reported studies, higher levels of stress were found to be related to elevated IL-6 and IL-1*β* in first and third trimesters and decreased IL-10 in first trimester [23]. However, stress was not related to IL-6 and TNF-*α* in a later study [33]. The only study that examined maternal fatigue and immune modulation during pregnancy found that elevated IL-1*β* at one week postpartum was related to fatigue at two weeks and one month postpartum. However, fatigue at 28 days postpartum was not related to IL-1*β* measured at the same time [34]. Moreover, while more depressive symptoms during pregnancy have been associated with higher levels of IL-6 and TNF-*α* [31, 33], other studies have not found this relationship [35].

Because of the detrimental influence of prenatal depression on maternal and child health, as well as the high prevalence of maternal stress and fatigue and their relationship to depression, and the lack of studies and inconsistent results regarding the immunomodulating effects of cytokines on these measures of maternal well-being, this study explored changes and relationships between perinatal stress, fatigue, depressive symptoms, and immune modulation as measured by cytokines. The research questions were (a) what changes occur in maternal stress, fatigue, depressive symptoms, and cytokines from late pregnancy to one month postpartum? and (b) what are the relationships between levels of stress, fatigue, depressive symptoms, and cytokines?

## 2. Methods

A psychoneuroimmunologic (PNI) model, which describes interrelationships between psychosocial factors, neuroimmunologic pathways, and health outcomes, was used to guide the study [36] The model hypothesizes that chronic stress leads to activation of the immune system, which may further lead to adverse health outcomes [37]. In other words, cytokines secreted in response to stress mediate the effects of psychosocial variables on health. In this study, maternal stress and fatigue were hypothesized to stimulate the immune system, measured by levels of serum cytokines, which in turn was hypothesized to contribute, with stress and fatigue, to maternal depressive symptoms.

### 2.1. Design

The study used a repeated measures correlational design. Data were collected when participants were at 36 weeks or greater gestation and at four to six weeks postpartum. The study was approved by the university's institutional review board, and informed, written consent was obtained from all participants.

### 2.2. Sample

The study included 46 pregnant women who were older than 17 years, without pregnancy complications, over 36 weeks of gestation, and understood English. Five women delivered before data collection or lost contact with the investigator and were thus dropped from the study. In addition, invalid or inadequate blood samples for prenatal cytokine analyses were provided by 3 participants. Twenty participants did not come to the postpartum visit and were lost to follow-up; 4 participants refused to provide blood samples and 2 participants did not complete the CES-D scale. This left 12 completed surveys and blood samples at both data collection points. Of these 12 participants, 58.3% were African American, 66.7% had a high school or higher education, 16.7% were married or partnered while 66.7% were single never married, 25% were full-time employed while 3.3% were part-time employed, 50% had a C-section, and 27.3% were primiparous. The participants' mean age was 28 (SD = 5.2) years. The mean gestational age of infants delivered by the sample participants was 39.3 (SD = 1.3) weeks and the mean birth weight was 3330.6 (SD = 515.2) grams. There were no differences in parity, race, marital status, educational level, employment, birth method, infant birth weight, and gestational age between participants who completed both prenatal and postnatal data collections and those who completed only the prenatal data collection.

### 2.3. Procedure

Participants were recruited from the prenatal clinic of a large, urban medical center. Pregnant women who met inclusion criteria were invited to participate in the study. Participants were asked to inform the investigators when they gave birth at which time an appointment for the 4 to 6 weeks postpartum data collection was made. The postpartum visit was scheduled to coincide with either a postpartum or well-child visit. If the participants did not inform the investigators that they had delivered, phone calls were made to the participants after their expected date of confinement. Participants were asked to complete a set of questionnaires and give 1 mL of blood at both data collection points.

### 2.4. Measures

*Maternal Stress*. The 10-item Perceived Stress Scale (PSS) was used to collect data about stress. The PSS-10 was shortened from the original 14-item PSS to measure the degree by which situations were appraised as stressful in one's life [38]. The PSS-10 is a 5-point (scores 0 to 4) response scale. A higher score on the PSS indicates higher levels of stress. The internal reliability and validity of the PSS-10 were supported in the scale-development study [38]. The Cronbach alpha for the PSS-10 at prenatal and postpartum intervals in the current study was .75 and .88, respectively. The median score of the PSS was used to divide participants into low and high stress groups in the current study; median scores were 20 and 17 in prenatal and postpartum period, respectively.

*Maternal Fatigue*. The study used a single item asking participants whether fatigue was a problem to them and if so how they rated their level of fatigue. Participants chose from not a problem (0), minor (1), or major (2) problem. The participants were categorized into yes (minor or major) and no (not a problem) fatigue groups for comparison.

*Maternal Depressive Symptoms*. Maternal depressive symptoms were measured with the CES-D. The CES-D is a 20-item, four-point (0–3) response scale [39]. Higher scores indicate more depressive symptoms. The CES-D has been used in samples of women during the perinatal period with good reliability [40]. The Cronbach alpha for the CES-D was .89 in both prenatal and postpartum women in the current study. A score of 16 is generally used as a cutoff for depression; this score was used to categorize participants into yes or no depression groups.

*Immune Function*. Immune function was measured by serum cytokine levels using 1 mL of blood. The serum of all blood samples was frozen at −20°C and not thawed until analysis; all samples were analyzed at the same time. Analyses were conducted using the Bioplex Cytokine Assay, a multiplex bead-based assay used to quantitate multiple cytokines in a single run in diverse matrices. In this study, 17 representative pro- and anti-inflammatory serum cytokines were analyzed, including IL-1*β*, IL-2, IL-4, IL-5, IL-6, IL-7, IL-8, IL-10, IL-12, IL-13, IL-17, macrophage inflammatory protein 1 alpha (MIP-1*α*), granulocyte macrophage colony stimulating factor (GM-CSF), IFN-*γ*, monocyte chemotactic protein (MCP-1), macrophage inflammatory protein 1-beta (MIP-1*β*), and TNF-*α*.

### 2.5. Data Analysis

All data were analyzed with SPSS 15.0. IL-2, IL-4, IL-6, IL-8, IL-10, IL-12, IL-13, IL-17, TNF-*α*, GM-CSF, and IFN-*γ* had out-of-range values for more than 30% of the available samples or could not be normalized after transformation; these cytokines were excluded from statistical analysis. All variables except prenatal and postnatal IL-1*β*, which were skewed, were normally distributed. All skewed variables were normalized by log procedures before inferential analyses were conducted. Descriptive statistics were used to analyze the participants' demographic information and level of measured variables. Paired *t*-test was used to test changes in variables from late pregnancy to the postpartum. Chi square or Fisher exact test was used to compare proportions between groups. Pearson correlations were used to test correlations between variables and independent *t*-test was used to compare cytokine levels between high-low stress, yes-no fatigue, and yes-no depression groups. Because of the small sample size, effect sizes were also computed. A significance level of .05 was set and 2-tailed test was used.

## 3. Results

### 3.1. Changes of Measured Variables from Prenatal to Postpartum

The results of changes in measured variables can be seen in Table 1. Participants although perceived higher stress than did general population of females in the USA, their difference in the level of perceived stress was not exceedingly high (*t* = 4.51, *P* = 0.001) [38]. Stress decreased from the prenatal periods to the postpartum period although the change was not statistically significant (*t* = 1.08, *P* = 0.31, *d* = 0.31). Mothers experienced fatigue about 750% and 83% of time in the prenatal and postpartum periods, respectively. In addition, 25% of mothers in the prenatal periods thought fatigue was a major problem. More mothers experienced depressive symptoms in the prenatal period (67%) than in postpartum (50%) although the difference was not statistically significant; however, during the prenatal period, mothers had significantly higher scores on the CES-D than in the postpartum (*t* = 2.96, *P* = 0.01, *d* = 0.62). Serum cytokine G-CSF increased from the prenatal to the postpartum period with a median high effect size (*t* = 2.13, *P* = 0.06, *d* = 0.62).

### 3.2. Relationships between Measured Variables

The relationships among variables can be seen in Table 2. Prenatal maternal stress, fatigue, and depression were intercorrelated. Prenatal stress had a medium but nonsignificant negative relationship with MCP (*r* = −0.37). Prenatal depressive symptoms had high but nonsignificant positive relationships with IL-7 (*r* = 0.51) but was significantly related with MIP-1*β* (*r* = 0.64). Fatigue had a medium but nonsignificant negative relationship with IL-5 (*r* = −0.47) and a positive relationship with MCP (*r* = 0.33). Levels of prenatal cytokines were not statistically different when participants were grouped as either low-high stress, yes-no fatigue, or yes-no depression.

Postnatal stress and depressive symptoms were strongly and positively correlated (*r* = 0.65) while depressive symptoms were related to fatigue with a median high effect size (*r* = 0.34). Postnatal stress was significantly related to postnatal depressive symptoms (*r* = 0.65). Postnatal stress had medium high positive relationships with postnatal MIP-1*β* (*r* = 0.41). Postnatal fatigue had medium nonsignificant positive relationships with postnatal G-CSF (*r* = 0.36) and MIP-1*β* (*r* = 0.30) whereas depressive symptoms had a medium nonsignificant relationship with IL-5 (*r* = 0.45). Levels of postnatal cytokines were not statistically different when participants were grouped as either low-high stress, yes-no fatigue, or yes-no depression.

## 4. Discussion

The study used a repeated measure design to examine relationships between stress, fatigue, depressive symptoms, and cytokines in late pregnancy and the early postpartum. Results showed that mothers experienced more depressive symptoms prenatally than postnatally and that maternal stress, fatigue, and depressive symptoms were intercorrelated. All cytokines analyzed in this study except for G-CSF, an anti-inflammatory cytokine that increased, did not show significant change from late pregnancy to postpartum.

Findings from this study are not different in some ways from those found in other studies. Concentrations of some cytokines remain stable during pregnancy. For example, levels of IL-1*β* in cervical mucus or vaginal washing fluid do not differ from the second trimester to the third trimester [41, 42]. Similar to IL-1*β*, levels of TNF-*α* remain stable during pregnancy [23, 29, 41, 43] with higher levels of TNF-*α* being related to preterm labor [44]. Another proinflammatory cytokine, IL-12, has similar activity; an increased level of IL-12 in amniotic fluid may cause oligohydramnios and further lead to preterm labor [45] or other pregnancy complications. In this study with all term labors, prenatal serum IL-1*β* was equal to its concentration in the postpartum. This result supports the hypothesis that preterm labor may be caused by infection/inflammatory processes that involve increased proinflammatory cytokines such as IL-1*β* and TNF-*α* [44, 46, 47], which were not seen in this study. In fact, this study also found positive correlations between pro- and anti-inflammatory cytokines such as IL-13 and TNF-*α*, and this finding is similar to findings in another study [44]. A balance of pro- and anti-inflammatory cytokines during late pregnancy as shown in this study may be essential to maintaining pregnancy, although the small sample size precludes drawing this conclusion.

The findings regarding MIP-1*α* require some explanation. MIP-1*α* is a chemokine. Chemokines, a family of small cytokines that have the ability to induce chemotaxis, play an important role in embryo implantation and labor. During embryo implantation, chemokines recruit and activate leukocytes and migrating trophoblast [48]. At term or in pathological conditions, several specific chemokines such as MIP-1*α* are hypothesized to recruit leukocytes inducing the further secretion of cytokines such as TNF-*α* and matrix metalloproteinases (MMPs). A cascade mechanism of activation of reproductive tissues then results in myometrial contractions, cervical ripening, and rupture of membranes [49]. Although no studies could be found focusing on MIP-1*β* specifically during pregnancy and the postpartum, it is possible that MIP-1*β*, like other chemokines, plays a role in activation of labor, explaining the findings in this study of medium but nonsignificant relationships between prenatal stress, depressive symptoms, and MIP-1*β* at time periods close to delivery. The immunological role of MIP-1*β* and its mediating role on stress and depression may be worth further investigation.

Similar to other study findings, the current study found that fatigue was experienced by many pregnant and postpartum women [8, 50] and was related to depressive symptoms both in the prenatal and postpartum periods [12, 19, 51, 52]. Interestingly only postpartum fatigue showed direct effects on depressive symptoms, even though there was no statistically significant difference in the percentage of mothers who experienced fatigue in the postpartum versus the prenatal period. Childcare responsibilities might explain some of the effects of postpartum fatigue on postpartum depressive symptoms. Since decreased energy or unexplainable fatigue is one of the diagnosing symptoms of depression [53] and perinatal fatigue influences both maternal and child health as well as pregnancy outcomes, fatigue [54], whether it is reported or not by pregnant or postpartum women, needs to be actively assessed. Mothers at high risk for fatigue should also be assessed for causes of fatigue such as anemia, thyroid conditions, and infection.

In the current study, occurrence and severity of depression were higher during pregnancy than in the postpartum period. Additionally, women who experienced more prenatal depressive symptoms experienced more postpartum depressive symptoms. Prenatal depression influences not only the pregnant woman but also her fetus and child [16], having potentially serious effects on women's health and on the health of their children. Yet, despite its influences, prenatal depression has not been fully studied. More studies, especially longitudinal and interventional studies, are needed to increase our knowledge about etiology, patterns, symptoms, factors, and management of prenatal depression. Moreover, the search for reliable biomarkers for women at risk for pre- and postnatal depression remains a priority.

## 5. Conclusion

The current study found that many mothers experienced fatigue and depressive symptoms and that mothers experienced higher levels of stress than women in the general population. Additionally, it was found that maternal stress, fatigue, depressive symptoms, and some aspects of immune modulation as measured by serum cytokines were related. However, given the limitations of the small sample size and missing data, conclusions about these findings are difficult to make. It is likely that the levels of pro- and anti-inflammatory cytokines found in this study are normal and that the balance seen is one that is necessary to maintain pregnancy. It is suggested that studies with larger samples using longitudinal designs from early pregnancy with multiethnicities be conducted. These studies should focus on biochemical markers that can be used to increase our knowledge about maternal immunity, physical and psychological well-being, and pregnancy outcomes.

## Figures and Tables

**Table 1:** Comparison of levels of cytokines and measured variables in late pregnancy and early postpartum.

	Prenatal			Postnatal			*t*	*p*	*r*	*d*
	Mean ± SD	Median	Range	Mean ± SD	Median	Range				
IL-1*β*	4.94 ± 11.47	.33	.04–39.26	6.55 ± 11.97	1.32	.15–34.00	1.29	.23	.15	.11
IL-5	1.38 ± 1.54	.67	.10–5.40	1.67 ± 1.05	1.55	.44–3.39	.77	.46	.56	.22
IL-7	10.10 ± 7.28	8.10	1.74–28.30	8.87 ± 3.64	9.23	4.02–16.75	−.67	.52	.48	.19
G-CSF	31.09 ± 15.46	32.70	7.38–54.30	46.94 ± 30.96	38.17	17.65–122.44	2.13	.06	.56	.62
MIP-1*β*	154.03 ± 90.45	141.23	38.74–350.50	186.53 ± 127.43	161.19	67.01–440.04	1.00	.34	.51	.29
MCP	34.51 ± 26.10	22.03	12.54–93.16	34.03 ± 22.19	33.78	7.12–90.22	−.09	.93	.72	.03
Stress	18.88 ± 3.98	18.00	14–26	17.08 ± 5.68	17.00	9–26	−1.08	.31	.30	.31
Depressive symptoms	20.50 ± 7.68	23.00	8–33	14.58 ± 6.37	16.00	2–22	−2.96	.01	.08	.62

	*n*		%	*N*		%	Chi/Fisher		*P*	

Fatigue							.37		.46	
Not a problem	3		25.0	2		16.7				
Minor	6		50.0	10		83.3				
Major	3		25.0	0		0				
Depressed	8		66.7	6		50.0	.55		.27	

**Table 2:** Correlations between measured variables and cytokines in late pregnancy and early postpartum.

	1	2	3	4	5	6	7	8	9	10	11	12	13	14	15	16	17
(1) Prestress	1.00																
(2) Predepression	.37	1.00															
(3) Prefatigue	.39	.46	1.00														
(4) Pre-IL1	.41	−.27	−.06	1.00													
(5) Pre-IL5	−.25	.15	−.47	.18	1.00												
(6) Pre-IL7	−.25	.51	.12	−.27	.24	1.00											
(7) Pre-GCSF	−.02	−.06	−.13	.24	−.07	−.28	1.00										
(8) Pre-MCP	−.37	.19	.33	−.14	.19	.39	−.09	1.00									
(9) Pre-MIP	.16	.64*	.12	−.30	.11	.21	.04	.25	1.00								
(10) Poststress	.33	.62*	.47	−.24	−.30	−.02	.02	.19	.73*	1.00							
(11) Postdepression	−.14	.53	.15	−.08	.19	.45	−.04	.34	.67*	.65*	1.00						
(12) Postfatigue	−.07	.21	.26	.06	.01	.20	.49	.14	.19	.05	.34	1.00					
(13) Post-IL1	.02	−.10	.29	.15	−.36	−.29	.05	−.23	−.40	.25	.12	−.11	1.00				
(14) Post-IL5	−.34	.24	−.10	−.08	.56	.73*	−.46	.19	.18	−.23	.45	.18	−.31	1.00			
(15) Post-IL7	−.54	−.06	−.16	−.32	.02	.48	−.17	.19	−.22	−.29	.10	.16	−.09	.34	1.00		
(16) Post-GCSF	−.07	−.08	.44	.28	−.17	−.32	.56	.13	−.32	.07	−.02	.36	.63*	−.36	−.20	1.00	
(17) Post-MCP	−.47	−.36	.13	−.12	−.09	−.10	.04	.72*	−.10	−.12	−.13	.08	−.20	−.16	.36	.19	1.00
(18) Post-MIP	.00	.05	−.21	−.30	−.39	−.08	.47	−.10	.51	.41	.24	.30	−.23	−.35	−.04	−.21	−.03

*P < .05, **P < .01.
